# Identifying lncRNA–miRNA–mRNA networks to investigate Alzheimer’s disease pathogenesis and therapy strategy

**DOI:** 10.18632/aging.102785

**Published:** 2020-02-07

**Authors:** Nana Ma, Changrui Tie, Bo Yu, Wei Zhang, Jun Wan

**Affiliations:** 1 Shenzhen Key Laboratory for Neuronal Structural Biology, Biomedical Research Institute, Shenzhen Peking University - The Hong Kong University of Science and Technology Medical Center, Shenzhen 518000, Guangdong Province, China; 2 Shenzhen Key Laboratory for Translational Medicine of Dermatology, Biomedical Research Institute, Shenzhen Peking University - The Hong Kong University of Science and Technology Medical Center, Shenzhen 518000, Guangdong Province, China; 3 Department of Dermatology, Peking University Shenzhen Hospital, Shenzhen 518000, Guangdong Province, China; 4 Division of Life Science, The Hong Kong University of Science and Technology, Kowloon, Hong Kong 999077, China

**Keywords:** Alzheimer’s disease (AD), ceRNA network, APP/PS1 mouse, RNA-sequencing, lncRNA

## Abstract

Alzheimer’s disease (AD), the most common cause of dementia, leads to neuronal damage and deterioration of cognitive functions in aging brains. There is evidence suggesting the participation of noncoding RNAs in AD-associated pathophysiology. A potential linkage between AD and lncRNA-associated competing endogenous RNA (ceRNA) networks has been revealed. Nevertheless, there are still no genome-wide studies which have identified the lncRNA-associated ceRNA pairs involved in AD. For this reason, deep RNA-sequencing was performed to systematically investigate lncRNA-associated ceRNA mechanisms in AD model mice (APP/PS1) brains. Our results identified 487, 89, and 3,025 significantly dysregulated lncRNAs, miRNAs, and mRNAs, respectively, and the most comprehensive lncRNA-associated ceRNA networks to date are constructed in the APP/PS1 brain. GO analysis revealed the involvement of the identified networks in regulating AD development from distinct origins, such as synapses and dendrites. Following rigorous selection, the lncRNA-associated ceRNA networks in this AD mouse model were found to be mainly involved in synaptic plasticity as well as memory (*Akap5*) and regulation of amyloid-β (Aβ)-induced neuroinflammation (*Klf4*). This study presents the first systematic dissection of lncRNA-associated ceRNA profiles in the APP/PS1 mouse brain. The identified lncRNA-associated ceRNA networks could provide insights that facilitate AD diagnosis and future treatment strategies.

## INTRODUCTION

Alzheimer’s disease (AD) was first described in 1906, which was recognized as a common cause of dementia and a major cause of death 70 years later [1]. Although AD has become a significant focus of research, the molecular mechanisms underlying its pathogenesis remain largely unknown [2]. At present, there are no effective prognostic biomarkers yet. Therefore, it is significant to identify not only the potential biomarkers for prediction of survival in this disease, but also the novel targets for prognosis improvement and guidance on the optimal individual treatment.

It was reported that 80% of the human genome is transcribed as noncoding RNAs (ncRNAs) [3], which can manipulate most of the potential biological functions [4]. Over the past two decades, it was demonstrated that ncRNAs, with their specific spatiotemporal expression patterns across various species, are extensively involved in numerous biological processes, including epigenetic regulation, chromatin remodeling, transcription control, and posttranscriptional processing [5]. More and more ncRNAs have been identified for their important roles in the pathogenesis of neurodegenerative disorders.

NcRNAs include microRNAs (miRNAs, ~20 nucleotides in length) and long noncoding RNAs (lncRNAs, larger than 200 nucleotides in length), which play significant roles in the regulation of various biological functions [6]. There is evidence that miRNAs and lncRNAs participate in AD pathophysiology, which includes the formation and development of β-amyloid (Aβ) plaques, neurofibrillary tangles, synaptic loss and neuronal death [7–9]. The ncRNAs have been detected by RNA-sequencing (RNA-seq) or microarrays in many organisms, which presents exciting implications for understanding the regulation of basic biological systems and pathophysiological conditions, as well as the development of new therapeutic treatment of many diseases [10].

LncRNAs modulate the nervous system in various biological dimensions, including epigenetic regulation [11, 12] and posttranscriptional regulation [13]. For example, β-site amyloid precursor protein (APP)-cleaving enzyme 1-antisense (BACE1-AS) stabilizes BACE1 RNA and promotes APP cleavage, which is actively involved in the pathogenesis of Alzheimer’s disease [6, 9]. Evf2 RNA controls adult hippocampal neurogenesis through regulating dynamic expression of downstream targets [14]. Both HOTAIR [6] and MALAT1 [15] RNAs are upregulated in brain tumors and promote tumor metastasis. Malat1 RNA regulates synaptogenesis in mouse hippocampal neurons through controlling gene expression [16].

Recent studies suggest that lncRNAs, circRNAs, pseudogenes and mRNAs may function as miRNA sponges [17–19]. They compete with each other through miRNA response elements (MREs) and modulate the progress of many diseases [20, 21] including AD [9, 6, 13]. In addition to post-transcriptional regulation, epigenetic modifications may also play a crucial role in AD pathogenesis [7, 22]. These refined and complicated regulatory networks may help to explain why the isolation of a single component (e.g., β-secretase and apolipoprotein E4) failed to fully account for the whole pathogenesis process of AD [23]. It suggests that the underlying mechanisms for the involvement of competing endogenous RNA (ceRNA) in AD remain to be determined.

**Figure 1:**
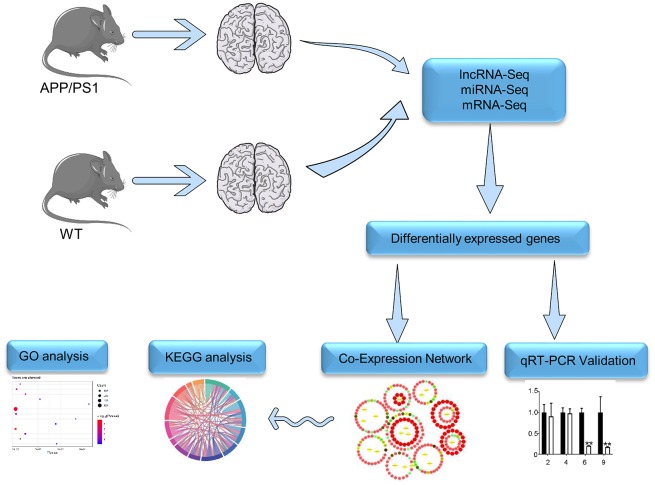
**The workflow of RNA-seq. Details of the methods used for mRNA-seq, miRNA-seq, and lncRNA-seq are described in Supplementary Materials.**

The rational strategy to gain insight into neurodegenerative diseases such as AD would make the study of lncRNA-associated ceRNA networks comprehensively. The elucidation of lncRNA-associated ceRNA networks in AD might help to develop new therapeutic targets for AD. In APP/PS1 mice which express APP695swe and PS1-dE9 mutations, Aβ can be detected in 6-month old mice, and then extracellular Aβ deposits in the cortex are apparent in 9-month old mice. Moreover, synaptic transmission and long-term potentiation are clearly impaired in 9-month old mice [24]. Thus, deep RNA-seq was performed in this study to find lncRNA-associated ceRNA networks in the brain of APP/PS1 mice together with the wild-type (WT) control mice at the 6- and 9-month-old stages. RNA-seq is widely used to determine the differential gene-expression profiles that underlie phenotypic differences [25, 26]. The data by RNA-seq identifies lncRNA-associated ceRNA networks in the APP/PS1 mouse model of AD (Figure 1), which can contribute to the development of new therapeutic targets and novel diagnostic methods for AD.

## RESULTS

### Overview of lncRNA and mRNA-seq data

A total of 1,145,853,722 raw reads were generated. 317,603,558 raw reads and 276,260,804 raw reads were generated for 6-month-old WT and APP/PS1 mice respectively, while 286,203,120 raw reads and 265,786,240 raw reads were generated for 9-month-old WT and APP/PS1 mice respectively. After discarding the reads with adapters, poly-N > 10%, or any other potential contaminants, 1,119,187,680 clean reads were obtained. 311,741,312 clean reads and 266,033,410 clean reads were obtained for 6-month-old WT and APP/PS1 mice respectively, while 278,938,832 clean reads and 262,474,126 clean reads were obtained for 9-month-old WT and APP/PS1 mice respectively. Both reference genome and gene model annotation files were downloaded directly from the genome website. An index of the reference genome was constructed with bowtie2 v2.2.8, and paired-end clean reads were aligned to the reference genome with HISAT2 v2.0.4 [27]. HISAT2 was run with “—rna-strandness RF,” while other parameters were set as default. Especially the mapping rates was 91.53% and 91.74% for APP/PS1 and WT mice, respectively. The transcripts were filtered out with coding potential prediction with the function of CNCI (Coding-Non-Coding-Index) (v2) [28], CPC (Coding Potential Calculator) (0.9-r2) [29], PfamScan (v1.3) [30], and PhyloCSF (phylogenetic codon substitution frequency) (v20121028) [31]. 9,299 lncRNAs (including 2,463 annotated lncRNA and 6,836 novel lncRNA) and 48,718 protein-coding transcripts (mRNA) were identified and used for subsequent analyses.

### Overview of miRNA-seq data

A total of 99,137,837 raw reads were generated. 28,672,205 raw reads and 20,760,197 were generated for 6-month-old WT and APP/PS1 mice respectively, while 23,016,993 raw reads and 26,688,442 raw reads were generated for 9-month-old WT and APP/PS1 mice respectively. After the removal of low quality and adapter sequences, 96,028,757 clean reads were obtained. 27,710,151 clean reads and 20,281,596 clean reads were obtained for 6-month-old WT and APP/PS1 mice respectively, while 22,393,001 clean reads and 25,644,009 clean reads were obtained for 9-month-old WT and APP/PS1 mice respectively. These clean reads were filtered by length (18–35 nucleotides), and a majority of the selected reads for both groups were 22 nucleotides in length. The selected reads were then mapped to the mouse reference sequence with Bowtie [32], and the mapping rate was 94.61% and 94.78% for APP/PS1 and WT mice respectively. Then, the mapped tags were then annotated and classified by alignment with noncoding small RNAs (including rRNA, tRNA, small nuclear RNA, and small nucleolar RNA), repeat-associated RNA, exon- and intron-associated RNAs in GenBank before sequenced in the miRBase v.20.0. In addition, both miREvo [33] and miRDeep2 [34] software was used to predict previously unidentified miRNAs. Ultimately, 1,411 mature miRNAs (1,312 known and 99 previously unknown) were detected and used for subsequent analyses.

### Differential expression analysis: APP/PS1 versus WT

**Figure 2:**
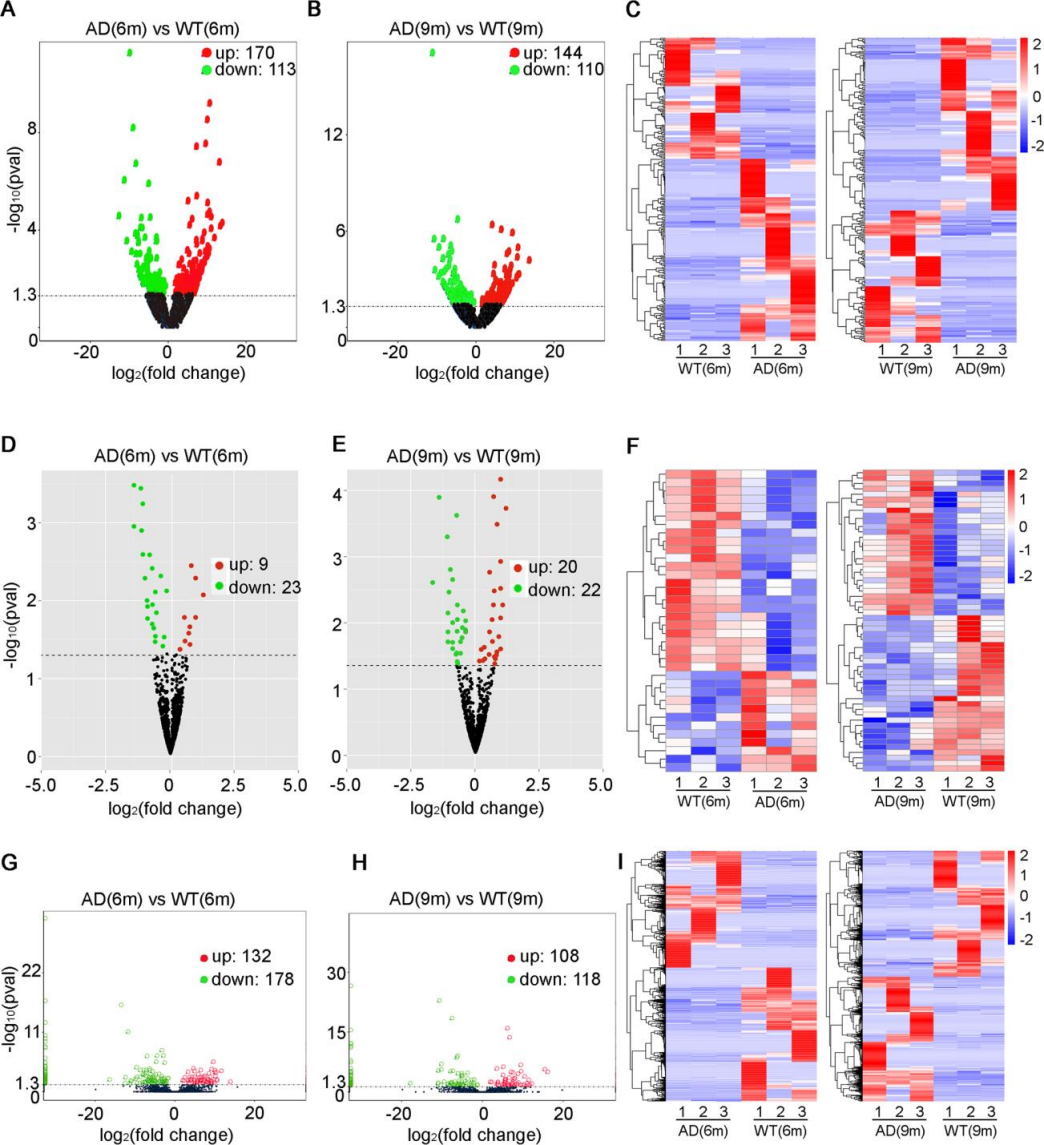
**Expression profiles of distinct RNAs.** (**A**–**C**) Expression profiles of lncRNAs. (**A**, **B**) In the volcano plots, green, red, and black points represent lncRNAs that were downregulated, upregulated, and not significantly different in APP/PS1 mice relative to wild-type (WT) control mice at 6 and 9 months, respectively. x-axis: log2 ratio of lncRNA expression levels between AD and WT. y-axis: false-discovery rate values (-log10 transformed) of lncRNAs, P<0.05 (**C**) Cluster analysis of expression of lncRNAs. Red and blue: increased and decreased expression at 6 and 9 months, respectively. Expression profiles are similarly shown for (**D**–**F**) miRNAs, p<0.04 and (**G**–**I**) mRNAs, q<0.05.

283 significantly dysregulated lncRNA transcripts (including 170 upregulated transcripts and 113 downregulated transcripts) were identified in the 6-month-old APP/PS1 mice (Figure 2A, Supplementary Table 1), while 254 significantly dysregulated lncRNA transcripts (including 144 upregulated transcripts and 110 downregulated transcripts) were identified in the 9-month-old APP/PS1 mice (Figure 2B, Supplementary Table 2). A heatmap was constructed to visualize the cluster analysis results of the lncRNAs expression (Figure 2C). Then based on the transcripts per million (TPM) values, 32 significantly dysregulated miRNAs were identified between the 6-month-old groups, which included 9 upregulated miRNAs and 23 downregulated miRNAs in APP/PS1 mice (Figure 2D, Supplementary Table 3). 42 miRNAs were significantly dysregulated between the 9-month-old groups, which included 20 upregulated miRNAs and 22 downregulated miRNAs in APP/PSI mice (Figure 2E, Supplementary Table 4). The cluster analysis of miRNAs expression was performed, and then a heatmap was generated (Figure 2F). Finally, the FPKM values (fragments per kilobase of exons per million fragments mapped) were used to estimate the expression levels of mRNA transcripts. 310 mRNA transcripts were significantly dysregulated, including 132 upregulated transcripts and 178 downregulated transcripts in APP/PS1 mice at 6 months (Figure 2G, Supplementary Table 5), while 226 mRNAs were significantly dysregulated, with 108 and 118 upregulated and downregulated in APP/PS1 mice at 9 months (Figure 2H, Supplementary Table 6). Once, cluster analysis for the expression of mRNAs was performed and a heatmap was generated (Figure 2I).

### qPCR validation

**Figure 3:**
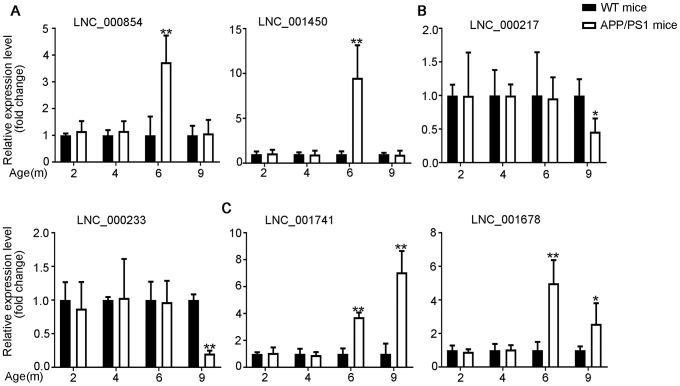
**Validation of expression of lncRNAs by using qPCR.** The identified differentially expressed transcripts (lncRNAs, miRNAs, and mRNAs) were divided into three groups. (**A**) 6yes9no group represents transcripts differential expressed at 6 months but not at 9 months; (**B**) 6no9yes group represents transcripts not differential expressed at 6 months but differential expressed at 9 months; (**C**) 6yes9yes group represents transcripts differential expressed at both 6 and 9 months. The expression of lncRNAs was quantified relative to *Gapdh* expression level by using the comparative cycle threshold (ΔCT) method. Data are presented as means ± SD (n = 3, *p < 0.05, **p < 0.01).

**Figure 4:**
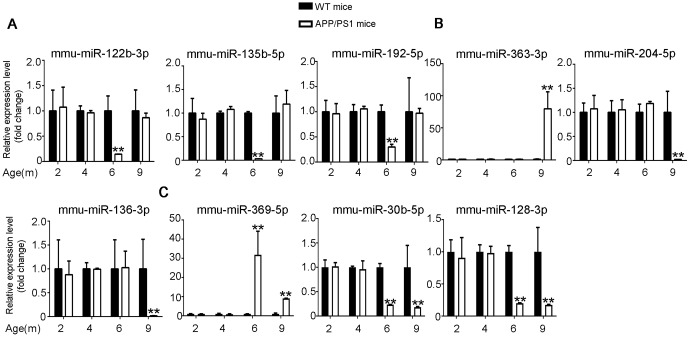
**Validation of miRNA expression by using qPCR.** (**A**) 6yes9no group, (**B**) 6no9yes group, and (**C**) 6yes9yes group. The expression levels of miRNAs were quantified relative to *U6* expression level by using the comparative cycle threshold (ΔCT) method. Data are presented as means ± SD (n = 3, *p < 0.05, **p < 0.01).

**Figure 5:**
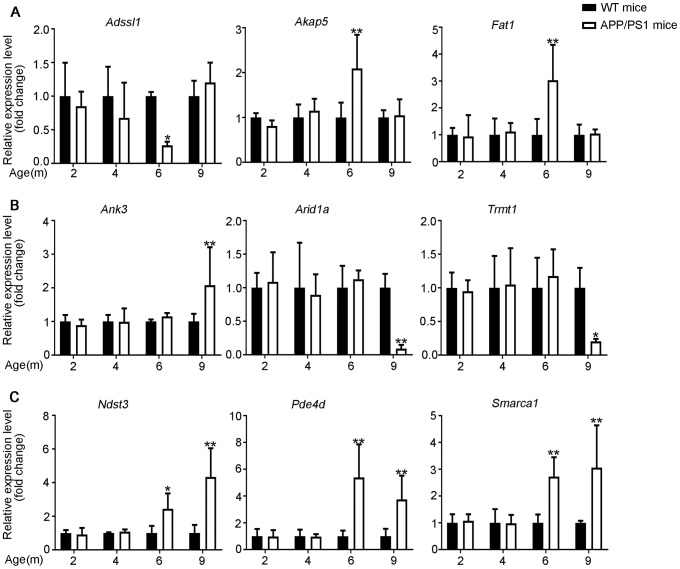
**Validation of mRNA expression by using qPCR.** (**A**) 6yes9no group, (**B**) 6no9yes group, and (**C**) 6yes9yes group. The mRNA expression was quantified relative to *Gapdh* expression level by using the comparative cycle threshold (ΔCT) method. Data are presented as means ± SD (n = 3, *p < 0.05, **p < 0.01).

The differential expression identified by RNA-seq experiments were confirmed with qPCR. 24 differentially expressed transcripts were randomly selected, including 6 lncRNAs, 9 miRNAs and 9 mRNAs. All of the selected transcripts were detected in the brain of 2–9-month-old APP/PS1 and WT mice. Besides, there was statistical difference between the two groups (Figures 3–5). Overall, the qPCR results were highly consistent with the RNA-seq data.

### Construction of lncRNA-associated ceRNA networks

**Figure 6:**
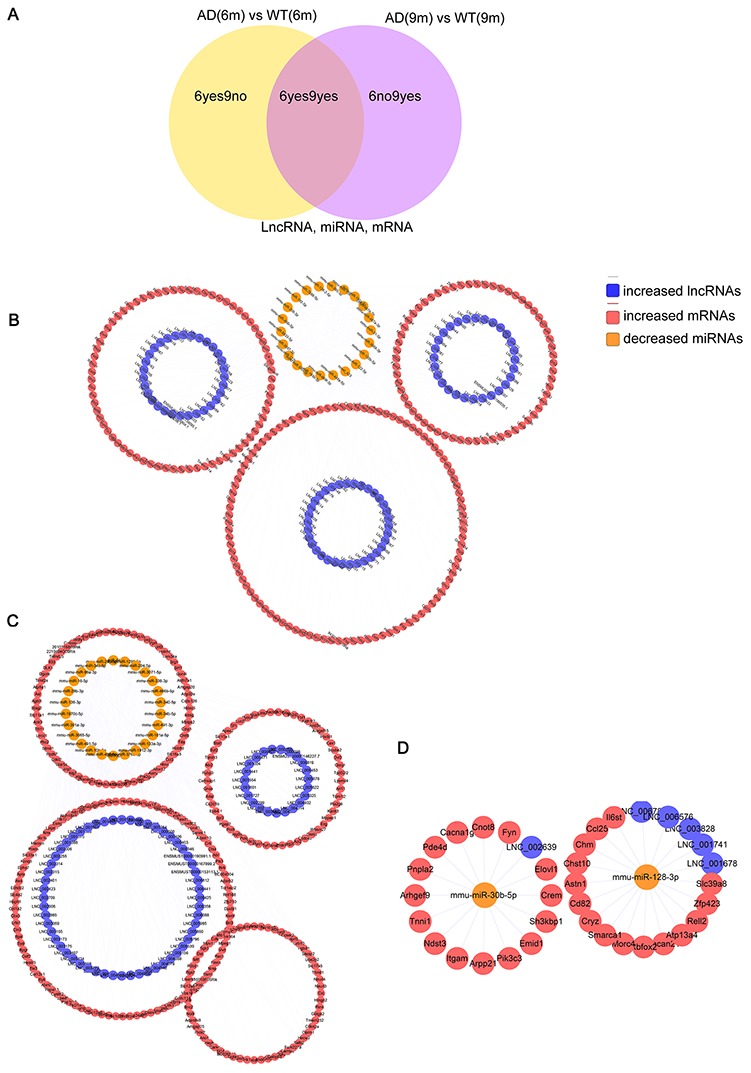
**The lncRNA-associated ceRNA networks in APP/PS1 mice.** CeRNA networks were constructed based on identified lncRNA–miRNA and miRNA–mRNA interactions. The networks include increased lncRNAs, decreased miRNAs, and increased mRNAs in APP/PS1 mice. (**A**) Grouping (**B**) 6yes9no group, (**C**) 6no9yes group, and (**D**) 6yes9yes group.

According to the ceRNA hypothesis, the ceRNAs can compete for the same MREs in regulatory networks. In this study, RNA-seq data were used to map ceRNA networks in the APP/PS1 brain for the first time. The differentially expressed transcripts (lncRNAs, miRNAs, and mRNAs) were split into three groups depending on the expression patterns. The 6yes9no group included transcripts differential expressed at 6 months but not differential expressed at 9 months of age, play a role in AD pathogenesis; The 6no9yes group included transcripts not differential expressed at 6 months but differential expressed at 9 months, participate in the development of AD; The 6yes9yes group included transcripts differential expressed at both 6 and 9 months, which contributed to in all stages of AD (Figure 6A).

**Figure 7:**
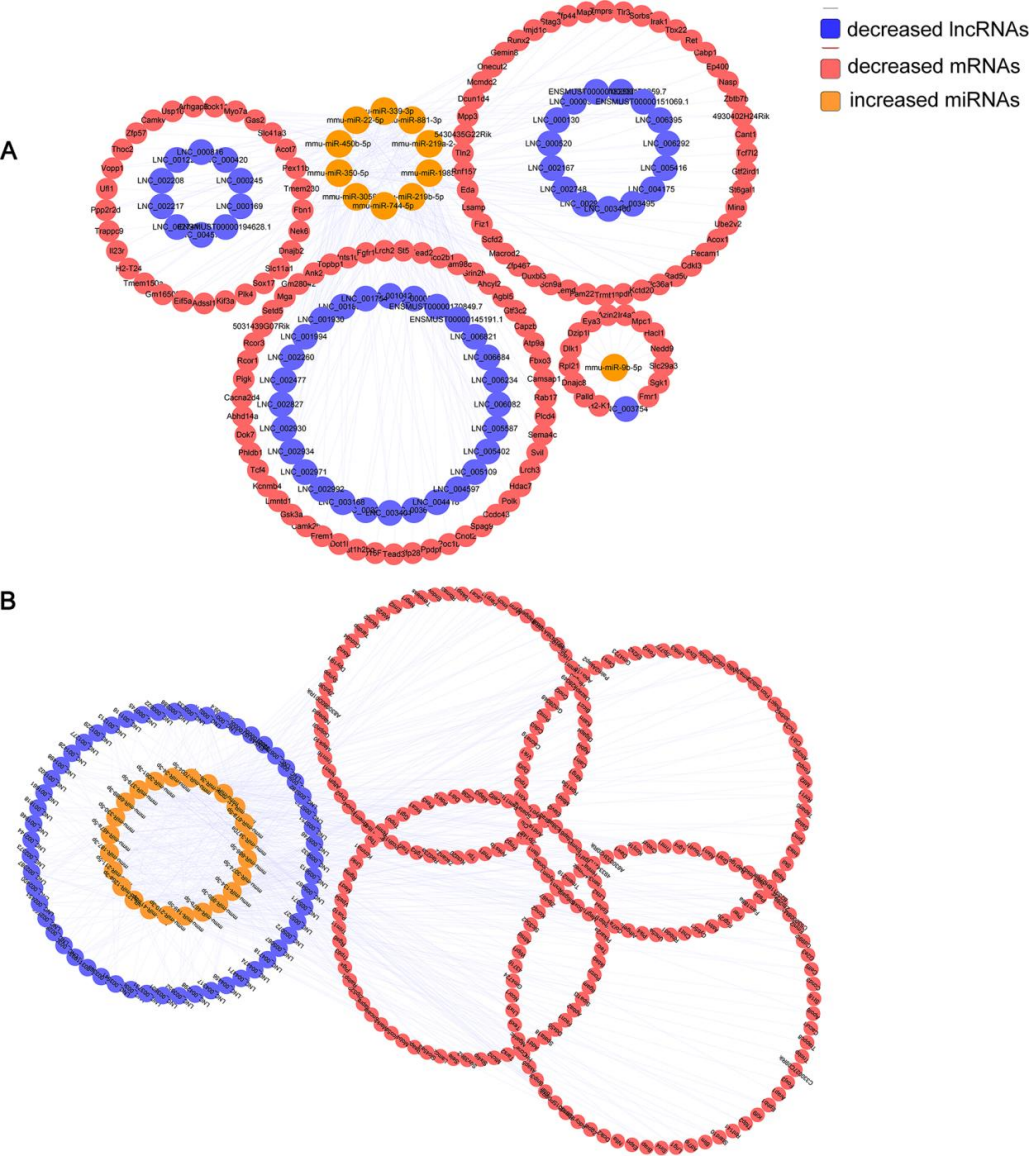
**Identified lncRNA-associated ceRNA networks in APP/PS1 mice.** The ceRNA networks were constructed based on identified lncRNA–miRNA and miRNA–mRNA interactions. The networks include decreased lncRNAs, increased miRNAs, and decreased mRNAs in APP/PS1 mice. (**A**) 6yes9no group, and (**B**) 6no9yes group.

6yes9no group included a total of 148 lncRNAs and 376 mRNAs that were differentially expressed and shared common MRE binding sites from 33 significantly dysregulated miRNAs (Supplementary Tables 7, 8). 6no9yes significantly dysregulated group included a total of 135 lncRNAs, 526 mRNAs, and 50 miRNAs (Supplementary Tables 9, 10). Besides, 7 lncRNAs, 31 mRNAs and 2 miRNA were included in 6yes9yes group (Supplementary Table 11). The ceRNA networks included both positive and negative regulation (Figures 6, 7). Figure 6 shows the increased lncRNAs, decreased miRNAs and increased mRNAs in APP/PS1 mice, while Figure 7 shows the decreased lncRNAs, increased miRNAs, and decreased mRNAs in APP/PS1 mice. It indicated potential critical RNA interactions involved in AD pathogenesis.

### Gene Ontology (GO) and Kyoto Encyclopedia of Genes and Genomes (KEGG) pathway analyses

**Figure 8:**
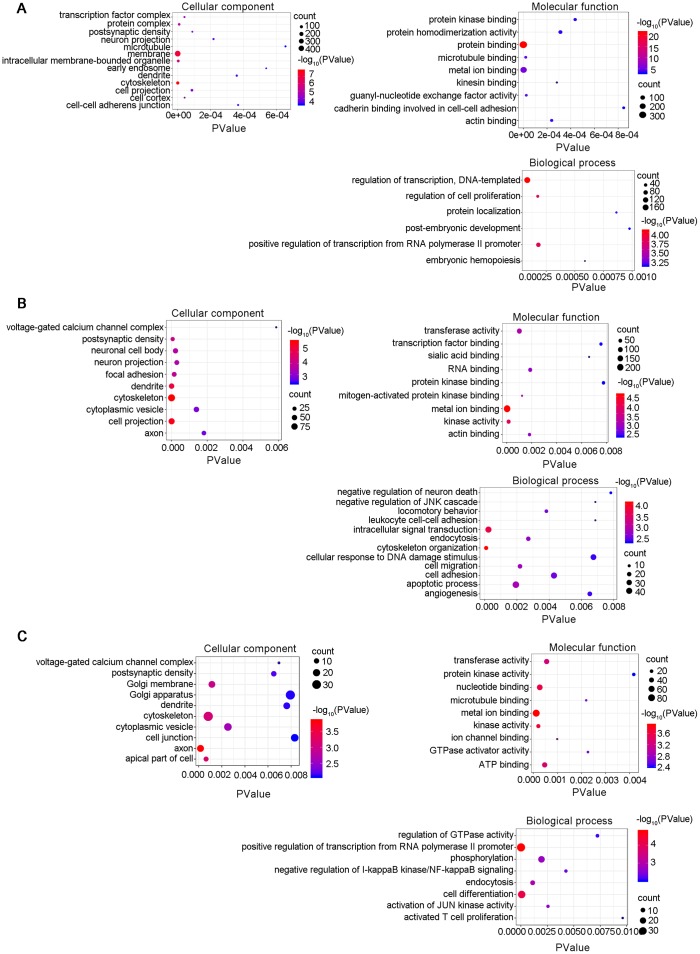
**Gene Ontology (GO) enrichment annotations of the pathological progression of AD: biological process, cellular component, molecular function.** The top terms were synapse (GO:0045202), cytoskeleton (GO:0005856), postsynaptic density (GO:0014069), cell-cell adherens junction (GO:0005913), dendrite (GO:0030425), axon (GO:0030424), and neuron projection (GO:0043005). (**A**) 6yes9no group, (**B**) 6no9yes group, and (**C**) 6yes9yes group. Significantly enriched GO pathways were defined as p values of <0.01. GO analysis was conducted with DAVID (https://david.ncifcrf.gov/summary.jsp) database.

A lncRNA-associated ceRNA network can alter the regulation of related mRNA-encoding genes. GO analyses were performed on the genes included in the networks identified here and several GO terms were found to be significantly enriched (Supplementary Tables 12–14). The GO terms included biological process (BP), cellular component (CC), and molecular function (MF), as shown in Figure 8. The top highly enriched terms were cytoskeleton (GO:0005856), postsynaptic density (GO:0014069), cell-cell adherens junction (GO:0005913) and dendrite (GO:0030425). A number of cognition-associated terms were also observed, such as axon (GO:0030424), synapse (GO:0045202), postsynaptic density (GO:0014069), intracellular signal transduction (GO:0035556), and neuron projection (GO:0043005). Noteworthy, the enriched GO terms for 6yes9no group were different from those for 6no9yes group, which suggested the expression changes in the functional genes during the progression of disease. For example, the Go pathways "transferase activity" was found in the "Molecular function" section of both B and C, but not in A of the Figure 8. It was explained that AD is characterized by the accumulation of intracellular and extracellular proteins, including the microtubule-associated protein Tau and the decomposition product of amyloid precursor protein β-amyloid Aβ. Tissue-type transglutaminase (tTG) is a calcium-dependent enzyme that catalyzes the cross-linking of proteins to generate isomeric peptides of the γ-glutamyl-ε-lysine structure (representing transferase activity). This covalent attachment results in protein aggregation and deposition due to its strong resistance to proteolysis. In previous studies it was demonstrated that the protein levels of tTG and isopeptides were increased in the brain of patients with advanced AD, and the activity increases noticeably with age. These findings suggest that tTG may be an important cause of abnormal protein accumulation in advanced AD pathology, which may not have been changed in the early stages [35, 36]. Consequently, 6yes9no group is not enriched in this pathway. In summary, the lncRNA-associated ceRNA networks might participate in the pathological progression of AD at distinct stages through different mechanisms. The establishment of these networks helps to investigate the functions of the key genes in AD and to guide determination of the regulatory mechanisms between the components of the ceRNA network.

**Figure 9:**
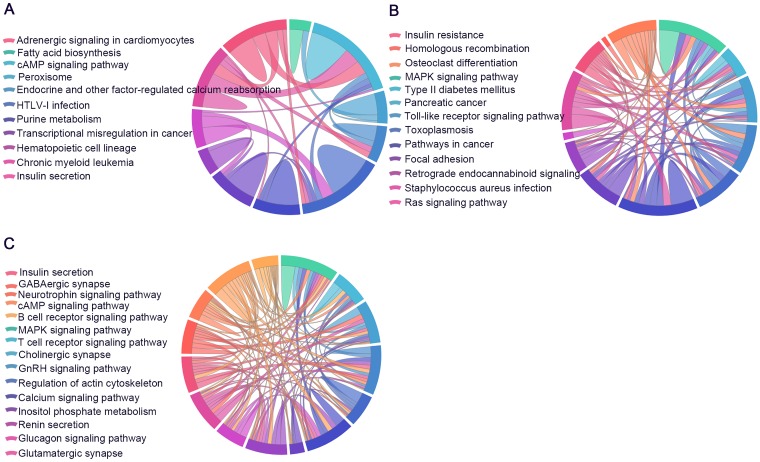
**Significantly enriched Kyoto Encyclopedia of Genes and Genomes (KEGG) and Reactome pathways.** The identified lncRNA-associated ceRNA-network genes participate in distinct aspects of AD pathology. (**A**) 6yes9no group, (**B**) 6no9yes group, and (**C**) 6yes9yes group. Significantly enriched KEGG pathways with p values of <0.05. Each line represents a gene, and the number of lines indicates the genes enriched. KEGG analysis was conducted with DAVID (https://david.ncifcrf.gov/summary.jsp) database.

KEGG pathway analysis was conducted to determine the signaling cascades related to the identified genes. By using p < 0.05 as the threshold value, a number of significantly enriched pathways were identified (Figure 9, Supplementary Tables 12–14), including neuroactive ligand-receptor interaction, AMP-activated protein kinase (AMPK) signaling, long-term potentiation, Hippo signaling, glutamatergic synapse, PI3K-Akt signaling, insulin secretion, focal adhesion and axon guidance.

### Association study

We selectively analyzed the data for lncRNAs and miRNAs of which the target genes significant differential expressed between APP/PS1 and WT mice (corrected p < 0.05). Additionally, we also selected lncRNAs and miRNAs of which the target genes showed enrichment in the mouse brain and were associated with AD. Analyses were performed to investigate the relationships between lncRNA-associated ceRNA networks and AD. For example, LNC_000854, LNC_001450, LNC_001451, LNC_001887, LNC_002205, LNC_002746, LNC_ 003197, LNC_003206, LNC_003458, LNC_004148, LNC_004514, LNC_004707 and LNC_006482 were identified as ceRNAs of mmu-miR-122-5p, which targets *Klf4*. The expression of *Klf4* was higher in AD mice than in WT mice. *Klf4* regulates amyloid-β (Aβ)-induced neuroinflammation and plays a potential role in not only oligomeric Aβ42-induced neurotoxicity but also the pathogenesis of Alzheimer's disease [37]. LNC_000033 was found to be a ceRNA of mmu-miR-128-2-5p, mmu-miR-135b-5p, mmu-miR-3097-3p, mmu-miR-31-5p, and mmu-miR-449a-5p, which target *Synpo. Synpo* was upregulated in the sequencing data. *Synpo* have been identified as crucial components in dendritic spine plasticity of the developing hippocampus. The disorder of *Synpo* expression affected the development of dendritic spines [38–42], which might also affect the early stages of AD. In addition, our analysis also revealed that miRNAs may act directly on their target genes. For example, *Grsf1*, targeted by mmu-miR-187-3p, mmu-miR-363-3p and mmu-miR-7004-5p, could be essential for the development of embryonic brain [43]. The additional results are listed in Supplementary Tables 7–11. Overall, it suggested that the identified lncRNA-associated ceRNA networks may be involved in the regulation of AD.

## DISCUSSION

AD is the most common neurodegenerative disease, of which the exact pathologic mechanism remains unknown. Although the traditional research tools and expertise detect some early brain changes of AD, additional research technologies remains necessary to fine-tune the accuracy of these tools. Recently, several studies on AD have focused on the epigenetic regulation of AD pathogenesis and identified the potential targets for therapy. lncRNAs expressed in brains have been reported to be contributory to the pathophysiology of AD [44, 45]. However, the roles of the lncRNAs in AD have remained mostly unknown.

MiRNAs are 22-nucleartide-long ncRNAs that can induce target gene silencing through complementary base-pairing with MREs on their 3ʹ UTRs and recruitment of RNA induced silencing complex (RISC) [46]. A total of 519 canonical miRNA genes have been identified in the human genome [47, 48]. There are roughly 70% of the identified miRNAs expressed in the provisional brain and in neurons [49], which suggests that miRNAs perform critical regulatory functions in the development of central nervous system (CNS), the formation of dendritic spine, neurite outgrowth, as well as neuronal differentiation and maintenance. The deregulation of miRNA is involved in neurodegenerative disorders such as AD and Parkinson’s disease (PD) [6], as well as in psychiatric disorders such as schizophrenia [50]. For example, let-7, miR-15a and miR-101 target APP, while miR-15a, miR-9 and miR-107 regulate BACE1 [8].

Over the past several years, the ceRNA hypothesis has been validated by numerous experiments. So far, ceRNA mechanisms and network construction have been mainly studied in the field of cancer research [51–54]. Only a small number of ceRNA interactions have been reported to be associated with neurodegenerative disorders. Recently, researchers have started to explore the ceRNA regulatory mechanism for specific neurodegenerative disorders in a systematic manner. Nevertheless, exciting advances have been made in our understanding of ceRNA interactions in neurodegenerative disorders. In our previous study, the lncRNA-ceRNA network was constructed in the brain of 12-month-old APP/PS1 mice [55]. As the sequencing depth and coverage limit the amount of generated sequences, the compositions of networks are slightly different. However, the key pathways in AD have been discovered. In order to not only explore changes of the ceRNA networks in the early stages of AD but also identify the early biomarkers, lncRNA-ceRNAs networks were constructed in the brain of 6- and 9-month-old APP/PS1 mice.

So far, it is the first comprehensive high-throughput sequencing analysis of the expression profiles of lncRNA, miRNA and mRNA in the APP/PS1 mouse model of AD. The dysregulated lncRNAs, miRNAs and mRNAs exhibited significant differential expression between AD and WT control groups, which suggested that these transcripts are associated with the pathogenesis of AD. For example, *Fndc3b* is essential for proliferation, adhesion, spreading and migration of nerve cells [56]. *Trappc9*, another differential expressed gene, plays a critical role in the development of human brain, possibly through its effect on NF-kappaB activation and protein trafficking in the postmitotic neurons of the cerebral cortex [57]. *Acsl6* mRNA is highly enriched in the brain and acsl6-/- mice demonstrate motor impairments, altered glutamate metabolism, increased astrogliosis and microglia activation [58]. Mmu-miR-376a-5p is a pancreatic islet-specific miRNA that regulates insulin secretion as an important pathway for the development of AD [59], while mmu-miR-134 regulates the development of cortical neurons [60]. The level of mmu-miR-29 is also substantially reduced in AD patients and it acts to regulate BACE1 expression [61, 62]. The qRT-PCR experiment confirmed the profiles from the high-throughput sequencing data, which indicated the reliability of the sequencing data.

In our study, RNA-seq was used to systematically analyze lncRNA, miRNA and mRNA profiles in the brain of 6- and 9-month-old APP/PS1 mice. The transcripts of 6yes9no group might participate in AD pathogenesis, of which the stability and specific expression could make them suitable as optimal biomarkers for AD. The transcripts in 6no9yes group might function in the development of AD. It is worth noting that the transcripts in 6yes9yes group may be involved in the disease at all stages, which suggests that focus on these transcripts could facilitate the development of lncRNA-based diagnostic tools and therapeutic strategies for AD. Overall, lncRNA and miRNA molecules have a potential to act as key regulators AD in different aspects. Both lncRNAs and protein-coding mRNAs function as ceRNAs and super-sponges to regulate the expression of miRNA. Therefore, we predicted the miRNA–mRNA and miRNA–lncRNA interaction with miRanda and constructed DElncRNA–DEmiRNA–DEmRNA triple networks for APP/PS1 and WT mouse brain. The selected lncRNA-associated ceRNA networks may facilitate new insights into AD and contribute novel treatments for the disease.

We performed GO enrichment and KEGG analysis of the genes in the ceRNA networks and identified not only a number of enriched terms relevant to the pathological process of AD, including cytoskeleton (GO:0005856), cell adhesion (GO:0005913), dendrite (GO:0030425), postsynaptic density (GO:0014069), axon (GO:0030424), synapse (GO:0045202), and neuron projection (GO:0043005), but also various pathways, including MAPK signaling, insulin secretion, Type II diabetes mellitus, cAMP signaling, Hippo signaling, focal adhesion, dopaminergic synapse, and PI3K-Akt signaling pathways. Analysis of the data revealed several lncRNA-associated ceRNA networks that participate in AD. *Akap5* contributes to synaptic plasticity mediated by NMDARs and AMPA-type glutamate receptors (AMPARs) and plays a critical role in the progression of AD [21, 63, 64]. One of these networks involves the gene *Akap5* and the ceRNAs, including LNC_000217, LNC_000233, LNC_000622, LNC_001498, LNC_001502, LNC_001818, LNC_ 002144, LNC_002373, LNC_002451, LNC_002620, LNC_003852, LNC_004317, LNC_005072, and LNC_005613. These ceRNAs have the potential to bind mmu-miR-679-5p, which targets *Akap5*. The APP/PS1 mice used in this study cannot represent the whole disease, which is primarily related to β-amyloid toxicity. Therefore, further research is necessary to better understand the regulation of these networks in AD.

Our research is just the beginning so that there remain many challenges and problems to be solved in the future. The cumulative evidence has helped to refine the dynamic ceRNA regulation [20], which reveals that various factors could contribute to the creation of miRNA and ceRNA hierarchies. These factors can be summarized as follows: miRNA target-site efficacy; shared MRE abundance; miRNA/ceRNA expression level and subcellular localization [21]; miRNA: target ratio; competition between rate-limiting molecules, such as Ago [63]; and advanced ceRNA hierarchy strategies. Moreover, attempt was made to decode the complexity of ceRNA regulatory networks. After the initial establishment of the ceRNA network, the biological mechanisms and functions mediated by the ceRNA mechanism should be predicted and verified by means of in vivo experiments in the future.

For a long time, there has been widespread recognition that ncRNAs are incapable to encode proteins [64]. Nevertheless, with the advancement of deep ribosome profiling sequencing (Ribo-Seq) technology, mass spectrometry and algorithms, a subset of ncRNA have been identified to encode peptides (<100 amino acids) or proteins, such as muscle-specific lncRNAs [65–67] and cancer-related lncRNA HOXB-AS3 [68]. At present, only few studies focused on Alzheimer's disease. Peptides/proteins encoded by ncRNAs might represent the drug targets or biomarkers for the prognosis of AD patients. Therefore, it’s significant to summarize the characteristics of peptides/proteins encoded by ncRNAs and their outlook for small molecule peptide drugs, drug targets and biomarkers.

In conclusion, the brain lncRNA-associated ceRNA profiles of APP/PS1 and WT mice were clarified. Our findings improve the current understanding of ceRNA biology and the regulatory roles of these RNAs in the pathogenesis of AD. These new networks reveal the potential biomarkers and may offer a promising target for the development of drugs to treat AD.

## MATERIALS AND METHODS

### Tissue preparation

WT and APP/PS1 mice [originally from The Jackson Laboratory; strain B6.Cg-Tg(APPswe, PSEN1dE9) 85Dbo/Mmjax [43]] were purchased from the Model Animal Research Center of Nanjing University. The mice were housed one per cage under standard conditions (25°C, 50% humidity, a 12-hour light/dark cycle, and specific-pathogen-free environment). The mice were provided with free access to the standard diet until they met the age requirements (6 and 9 months). 3 AD and 3 wild-type male mice born in the same litter were used as experimental and control groups, of which cerebral cortex samples were collected for RNA-seq. All animal experiments were performed in accordance with animal use protocols approved by the Committee for the Ethics of Animal Experiments, Shenzhen Peking University, the Hong Kong University of Science and Technology Medical Center (SPHMC) (protocol number 2011-004).

### RNA extraction and qualification

Total RNA from each sample was isolated with TRIzol reagent (Invitrogen) according to the manufacturer’s instructions and separated on 1% agarose gels to assess RNA degradation and contamination. RNA purity was measured with a NanoPhotometer spectrophotometer (IMPLEN, CA, USA). RNA concentration was measured with a Qubit RNA Assay Kit in a Qubit 2.0 Fluorometer (Life Technologies, CA, USA). RNA integrity was evaluated with the RNA Nano 6000 Assay Kit of a Bioanalyzer 2100 System (Agilent Technologies, CA, USA).

### RNA-seq

Details of the mRNA-seq, miRNA-seq, and lncRNA-seq methods are described in Supplementary Materials.

### Expression analysis

We calculated the FPKM values of transcripts by using Cuffdiff (v.2.1.1) to evaluate the expression levels of protein-coding genes and lncRNA in each sample [69]. The expression levels of miRNAs were estimated as TPM values as described [69]. Transcripts with p values less than 0.05 were regarded as being differentially expressed between APP/PS1 and WT mice. Normalized expression = (mapped reads)/(total reads) * 1,000,000.

### CeRNA network analysis

The expression levels of lncRNAs, miRNAs, and mRNAs differed significantly between APP/PS1 and WT mice. We searched the sequences of the lncRNAs and mRNAs to identify potential MREs. We used miRanda (http://www.microrna.org/microrna/) to predict miRNA-binding seed-sequence sites, and the presence of the same miRNA-binding sites in both lncRNAs and mRNAs indicated potential lncRNA–miRNA–mRNA interaction.

### GO annotations and KEGG pathway analyses

The DAVID (https://david.ncifcrf.gov/summary.jsp) database was used to analyze lncRNA-miRNA-enriched genes. GO and KEGG terms with p values less than 0.05 were considered significantly enriched.

### Construction of lncRNA-associated ceRNA networks

The lncRNA-associated ceRNA networks were constructed and visually displayed by using Cytoscape software V3.5.0 (San Diego, CA, USA) based on the analysis of high-throughput sequencing data, as described above. In the figures, distinct shapes and colors are used to represent different RNA types, and regulatory relationships.

### Real-time qPCR validation

Total RNA was extracted by using TRIzol reagent (Sigma) according to the manufacturer’s protocol. RNA quantity was measured by using a NanoDrop 2000 (Thermo Fisher Scientific). Quantitative RT-PCR was performed by using the GoScript^TM^ Reverse Transcription System (Promega), in a C1000 Thermal Cycler (Bio-Rad). The glyceraldehyde-3-phosphate dehydrogenase gene (*Gapdh*) and U6 were was used as an internal control. Relative gene-expression levels were calculated using the 2^−ΔCt^ method (n=3).

### Statistical analysis

Two normally distributed groups were compared by using *t* tests. Parameters for the high-throughput sequencing-related data were calculated, and statistical computing was performed by using R software. All data are expressed as means ± SD; a value of p < 0.05 was considered statistically significant.

### Data access

All raw and processed sequencing data generated in this study have been submitted to the NCBI Gene Expression Omnibus (GEO; http://www.ncbi.nlm.nih.gov/geo/) under accession number GSE132177.

## Supplementary Material

Supplementary Methods and References

Supplementary Table 1

Supplementary Table 2

Supplementary Table 3

Supplementary Table 4

Supplementary Table 5

Supplementary Table 6

Supplementary Table 7

Supplementary Table 8

Supplementary Table 9

Supplementary Table 10

Supplementary Table 11

Supplementary Table 12

Supplementary Table 13

Supplementary Table 14
