# Supplementary material for: Identifying lncRNA–miRNA–mRNA networks to investigate Alzheimer’s disease pathogenesis and therapy strategy
Source: Aging (Albany NY). 2020 Feb 7;12(3):2897–920. doi: 10.18632/aging.102785 (PMC7041741; doi:10.18632/aging.102785)
Supplement: Supplementary Methods and References [file aging-12-102785-s012..pdf]

SUPPLEMENTARY METHODS

MIRNA Methods

RNA isolation, quantification and qualification

RNA degradation and contamination was monitored on 1% agarose gels. RNA purity was checked using the Nano Photometer® spectrophotometer (IMPLEN, CA, USA). RNA concentration was measured using Qubit® RNA Assay Kit in Qubit® 2.0 Flurometer (Life Technologies, CA, USA). RNA integrity was assessed using the RNA Nano 6000 Assay Kit of the Bioanalyzer 2100 system (Agilent Technologies, CA, USA).

Library preparation for small RNA sequencing

A total amount of 3 μg total RNA per sample was used as input material for the small RNA library. Sequencing libraries were generated using NEBNext® Multiplex Small RNA Library Prep Set for Illumina® (NEB, USA.) following manufacturer's recommendations and index codes were added to attribute sequences to each sample. Briefly, NEB 3' SR Adaptor was directly, and specifically ligated to 3' end of miRNA, siRNA and piRNA. After the 3' ligation reaction, the SR RT Primer hybridized to the excess of 3' SR Adaptor (that remained free after the 3' ligation reaction) and transformed the single-stranded DNA adaptor into a double-stranded DNA molecule. This step is important to prevent adaptor-dimer formation, besides, dsDNAs are not substrates for ligation mediated by T4 RNA Ligase 1 and therefore do not ligate to the 5´ SR Adaptor in the subsequent ligation step. 5´ends adapter was ligated to 5´ends of miRNAs, siRNA and piRNA. Then first strand cDNA was synthesized using M-MuLV Reverse Transcriptase (RNase H–). PCR amplification was performed using LongAmp Taq 2X Master Mix, SR Primer for illumina and index (X) primer. PCR products were purified on a 8% polyacrylamide gel (100V, 80 min). DNA fragments corresponding to 140~160 bp (the length of small noncoding RNA plus the 3' and 5' adaptors) were recovered and dissolved in 8 μL elution buffer. At last, library quality was assessed on the Agilent Bioanalyzer 2100 system using DNA High Sensitivity Chips.

Clustering and sequencing

The clustering of the index-coded samples was performed on a cBot Cluster Generation System using TruSeq SR Cluster Kit v3-cBot-HS (Illumia) according to the manufacturer's instructions. After cluster generation, the library preparations were sequenced on an Illumina Hiseq 2500/2000 platform and 50bp.

Data analysis

Quality control

Raw data (raw reads) of fastq format were firstly processed through custom perl and python scripts. In this step, clean datas(clean reads) were obtained by removing reads containing ploy-N, with 5′ adapter contaminants, without 3′ adapter or the insert tag, containing ploy A or T or G or C and low quality reads from raw data. At the same time, Q20, Q30, and GC-content of the raw data were calculated. Then, chose a certain range of length from clean reads to do all the downstream analyses.

Reads mapping to the reference sequence

The small RNA tags were mapped to reference sequence by Bowtie (Langmead et al. 2009) without mismatch to analyze their expression and distribution on the reference.

Known miRNA alignment

Mapped small RNA tags were used to looking for known miRNA. miRBase20.0 was used as reference, modified software mirdeep2 (Friedlander et al. 2011) and srna-tools-cli were used to obtain the potential miRNA and draw the secondary structures. Custom scripts were used to obtain the miRNA counts as well as base bias on the first position of identified miRNA with certain length and on each position of all identified miRNA respectively.

Remove tags from these sources

To remove tags originating from protein-coding genes, repeat sequences, rRNA, tRNA, snRNA, and snoRNA, small RNA tags were mapped to RepeatMasker, Rfam database or those types of datas from the specified species itself.

Novel miRNA prediction

The characteristics of hairpin structure of miRNA precursor can be used to predict novel miRNA. The available software miREvo (Wen et al. 2012) and mirdeep2 (Friedlander et al. 2011) were integrated to predict novel miRNA through exploring the secondary structure, the Dicer cleavage site and the minimum free energy of the small RNA tags unannotated in the former steps. At the same time, custom scripts were used to obtain the identified miRNA counts as well as base bias on the first position with certain length and on each position of all identified miRNA respectively.

Small RNA annotation summary

Summarizing all alignments and annotations obtained before. In the alignment and annotation before, some small RNA tags may be mapped to more than one category. To make every unique small RNA mapped to only one annotation, we follow the following priority rule: known miRNA > rRNA > tRNA > snRNA > snoRNA > repeat > gene > NAT-siRNA > gene > novel miRNA > ta-siRNA. The total rRNA proportion was used a marker as sample quality indicator. Usually it should be less than 60% in plant samples and 40% in animal samples as high quality.

miRNA editing analysis

Position 2~8 of a mature miRNA were called seed region which were highly conserved. The target of a miRNA might be different with the changing of nucleotides in this region. In our analysis pipeline, miRNA which might have base edit could be detected by aligning all the sRNA tags to mature miRNA, allowing one mismatch.

miRNA family analysis

Exploring the occurrence of miRNA families identified from the samples in other species. In our analysis pipeline, known miRNA used miFam.dat (http://www.mirbase.org/ftp.shtml) to look for families; novel miRNA precursor was submitted to Rfam (http://rfam.sanger.ac.uk/search/) to look for Rfam families.

Target gene prediction

Predicting the target gene of miRNA was performed by miRanda (Enright et al, 2003) for animals.

Quantification of miRNA

miRNA expression levels were estimated by TPM (transcript per million) through the following criteria (Zhou et al. 2010): Normalization formula: Normalized expression = mapped readcount/Total reads*1000000.

Differential expression of miRNA

For the samples with biological replicates: Differential expression analysis of two conditions/groups was performed using the DESeq R package (1.8.3). The P-values was adjusted using the Benjamini and Hochberg method. Corrected P-value of 0.05 was set as the threshold for significantly differential expression by default.

LNCRNA and MRNA Methods

RNA isolation, quantification and qualification

RNA degradation and contamination was monitored on 1% agarose gels. RNA purity was checked using the Nano Photometer® spectrophotometer (IMPLEN, CA, USA). RNA concentration was measured using Qubit® RNA Assay Kit in Qubit® 2.0 Flurometer (Life Technologies, CA, USA). RNA integrity was assessed using the RNA Nano 6000 Assay Kit of the Bioanalyzer 2100 system (Agilent Technologies, CA, USA).

Library preparation for lncRNA sequencing

A total amount of 3 µg RNA per sample was used as input material for the RNA sample preparations. Firstly, ribosomal RNA was removed by Epicentre Ribo-zero™ rRNA Removal Kit (Epicentre, USA), and rRNA free residue was cleaned up by ethanol precipitation. Subsequently, sequencing libraries were generated using the rRNA-depleted RNA by NEBNext® Ultra™ Directional RNA Library Prep Kit for Illumina® (NEB, USA) following manufacturer's recommendations. Briefly, fragmentation was carried out using divalent cations under elevated temperature in NEBNext First Strand Synthesis Reaction Buffer(5X). First strand cDNA was synthesized using random hexamer primer and M-MuLV Reverse Transcriptase(RNaseH-). Second strand cDNA synthesis was subsequently performed using DNA Polymerase I and RNase H. In the reaction buffer, dNTPs with dTTP were replaced by dUTP. Remaining overhangs were converted into blunt ends via exonuclease/polymerase activities. After adenylation of 3′ ends of DNA fragments, NEBNext Adaptor with hairpin loop structure were ligated to prepare for hybridization. In order to select cDNA fragments of preferentially 150~200 bp in length, the library fragments were purified with AMPure XP system (Beckman Coulter, Beverly, USA). Then 3 µl USER Enzyme (NEB, USA) was used with size-selected, adaptor-ligated cDNA at 37° C for 15 min followed by 5 min at 95°C before PCR. Then PCR was performed with Phusion High-Fidelity DNA polymerase, Universal PCR primers and Index (X) Primer. At last, products were purified (AMPure XP system) and library quality was assessed on the Agilent Bioanalyzer 2100 system.

Clustering and sequencing

The clustering of the index-coded samples was performed on a cBot Cluster Generation System using TruSeq PE Cluster Kit v3-cBot-HS (Illumia) according to the manufacturer's instructions. After cluster generation,

the libraries were sequenced on an Illumina Hiseq 4000 platform and 150 bp paired-end reads were generated.

Data analysis

Quality control

Raw data(raw reads) of fastq format were firstly processed through in-house perl scripts. In this step, clean data(clean reads) were obtained by removing reads containing adapter, reads on containing ploy-N and low quality reads from raw data. At the same time, Q20, Q30 and GC content of the clean data were calculated. All the down stream analyses were based on the clean data with high quality.

Mapping to the reference genome

Reference genome and gene model annotation files were downloaded from genome website directly. Index of the reference genome was built using bowtie2 v2.2.8 and paired-end clean reads were aligned to the reference genome using HISAT2 (Langmead B. et al.) v2.0.4. HISAT2 was run with '--rna-strandness RF', other parameters were set as default.

Transcriptome assembly

The mapped reads of each sample were assembled by StringTie (v1.3.1) (Mihaela Pertea.et al. 2016) in a reference-based approach. StringTie uses a novel network flow algorithm as well as an optional de novo assembly step to assemble and quantitate full-length transcripts representing multiple splice variants for each gene locus.

Coding potential analysis

CNCI

CNCI (Coding-Non-Coding-Index) (v2) profiles adjoining nucleotide triplets to effectively distinguish protein-coding and non-coding sequences independent of known annotations (Sun et al. 2013). We use CNCI with default parameters.

CPC

CPC (Coding Potential Calculator) (0.9-r2) mainly through assess the extent and quality of the ORF in a transcript and search the sequences with known protein sequence database to clarify the coding and non-coding transcripts (Kong et al. 2007). We used the NCBI eukaryotes' protein database and set the e-value '1e-10' in our analysis.

Pfam-sca

We translated each transcript in all three possible frames and used Pfam Scan (v1.3) to identify occurrence of any of the known protein family domains documented in the Pfam database (release 27; used both Pfam A and Pfam B) (Punta et al. 2012). Any transcript with a Pfam hit would be excluded in following steps. Pfam searches use default parameters of -E 0.001 --domE 0.001 (Bateman et al. 2002).

PhyloCSF

PhyloCSF (phylogenetic codon substitution frequency) (v20121028) examines evolutionary signatures characteristic to alignments of conserved coding regions, such as the high frequencies of synonymous codon substitutions and conservative amino acid substitutions, and the low frequencies of other missense and non-sense substitutions to distinguish protein-coding and non-coding transcripts (Lin et al. 2011). We build multi-species genome sequence alignments and run phyloCSF with default parameters. Transcripts predicted with coding potential by either/all of the four tools above were filtered out, and those without coding potential were our candidate set of lncRNAs.

Conservative analysis

Phast (v1.3) is a software package contains much of statistical programs, most used in phylogenetic analysis (Siepel et al. 2005), and phastCons is a conservation scoring and identificating program of conserved elements. We used phyloFit to compute phylogenetic models for conserved and non-conserved regions among species and then gave the model and HMM transition parameters to phyloP to compute a set of conservation scores of lncRNA and coding genes.

Quantification of gene expression level

Cuffdiff (v2.1.1) was used to calculate FPKMs of both lncRNAs and coding genes in each sample (Trapnell C. et al. 2010). Gene FPKMs were computed by summing the FPKMs of transcripts in each gene group. FPKM means fragments per kilo-base of exon per million fragments mapped, calculated based on the length of the fragments and reads count mapped to this fragment.

Differential expression analysis

The Ballgown suite includes functions for interactive exploration of the transcriptome assembly, visualization of transcript structures and feature-specific abundances for each locus, and post-hoc annotation of assembled features to annotated features (Alyssa C. Frazee et al. 2014). Transcripts with an P-adjust <0.05 were assigned as differentially expressed. Cuffdiff provides statistical routines for determining differential expression in digital transcript or gene expression data using a model based on the negative binomial distribution (Trapnell C.

et al. 2010). Transcripts with an P-adjust <0.05 were assigned as differentially expressed.

Supplementary References

1. Anders S, Huber W. Differential expression analysis for sequence count data. Genome Biol. 2010; 11:R106. https://doi.org/10.1186/gb-2010-11-10-r106 PMID:20979621

2. Alyssa C. Frazee, Geo Pertea, Andrew E. Jaffe, Ben Langmead, Steven L. Salzberg

3. Enright AJ, John B, Gaul U, Tuschl T, Sander C, Marks DS. MicroRNA targets in Drosophila. Genome Biol. 2003; 5:R1. https://doi.org/10.1186/gb-2003-5-1-r1 PMID:14709173

4. Friedländer MR, Mackowiak SD, Li N, Chen W, Rajewsky N. miRDeep2 accurately identifies known and hundreds of novel microRNA genes in seven animal clades. Nucleic Acids Res. 2012; 40:37–52. https://doi.org/10.1093/nar/gkr688 PMID:21911355

5. Leek JT. Flexible analysis of transcriptome assemblies with Ballgown. bioRxiv. 2014.

6. Langmead B, Trapnell C, Pop M, Salzberg SL. Ultrafast and memory-efficient alignment of short DNA sequences to the human genome. Genome Biol. 2009; 10:R25. https://doi.org/10.1186/gb-2009-10-3-r25 PMID:19261174

7. Kanehisa M, Araki M, Goto S, Hattori M, Hirakawa M, Itoh M, Katayama T, Kawashima S, Okuda S, Tokimatsu T, Yamanishi Y. KEGG for linking genomes to life and the environment. Nucleic Acids Res. 2008; 36:D480–84. https://doi.org/10.1093/nar/gkm882 PMID:18077471

8. Langfelder P, Horvath S. WGCNA: an R package for weighted correlation network analysis. BMC Bioinformatics. 2008; 9:559. https://doi.org/10.1186/1471-2105-9-559 PMID:19114008

9. Mao X, Cai T, Olyarchuk JG, Wei L. Automated genome annotation and pathway identification using the KEGG Orthology (KO) as a controlled vocabulary. Bioinformatics. 2005; 21:3787–93. https://doi.org/10.1093/bioinformatics/bti430 PMID:15817693

10. McKenna A, Hanna M, Banks E, Sivachenko A, Cibulskis K, Kernytsky A, Garimella K, Altshuler D, Gabriel S, Daly M, DePristo MA. The Genome Analysis Toolkit: a MapReduce framework for analyzing next-generation DNA sequencing data. Genome Res. 2010; 20:1297–303. https://doi.org/10.1101/gr.107524.110 PMID:20644199

11. Siepel A, Bejerano G, Pedersen JS, Hinrichs AS, Hou M, Rosenbloom K, Clawson H, Spieth J, Hillier LW, Richards S, Weinstock GM, Wilson RK, Gibbs RA, et al. Evolutionarily conserved elements in vertebrate, insect, worm, and yeast genomes. Genome Res. 2005; 15:1034–50.

    https://doi.org/10.1101/gr.3715005 PMID:16024819

12. Storey JD. The positive false discovery rate: A Bayesian interpretation and the q-value. Ann Stat. 2003; 31:2013–35. https://doi.org/10.1214/aos/1074290335

13. Trapnell C, Williams BA, Pertea G, Mortazavi A, Kwan G, van Baren MJ, Salzberg SL, Wold BJ, Pachter L. Transcript assembly and quantification by RNA-Seq reveals unannotated transcripts and isoform switching during cell differentiation. Nat Biotechnol. 2010; 28:511–5. https://doi.org/10.1038/nbt.1621 PMID:20436464

14. Wang L, Feng Z, Wang X, Wang X, Zhang X. DEGseq: an R package for identifying differentially expressed genes from RNA-seq data. Bioinformatics. 2010; 26:136–38. https://doi.org/10.1093/bioinformatics/btp612 PMID:19855105

15. Wen M, Shen Y, Shi S, Tang T. miREvo: an integrative microRNA evolutionary analysis platform for next-generation sequencing experiments. BMC Bioinformatics. 2012; 13:140. https://doi.org/10.1186/1471-2105-13-140 PMID:22720726

16. Wu HJ, Ma YK, Chen T, Wang M, Wang XJ. PsRobot: a web-based plant small RNA meta-analysis toolbox. Nucleic Acids Res. 2012; 40:W22–8. https://doi.org/10.1093/nar/gks554 PMID:22693224

17. Young MD, Wakefield MJ, Smyth GK, Oshlack A. goseq: Gene Ontology testing for RNA-seq datasets. (goseq) 2010.

18. Zhou L, Chen J, Li Z, Li X, Hu X, Huang Y, Zhao X, Liang C, Wang Y, Sun L, Shi M, Xu X, Shen F, et al. Integrated profiling of microRNAs and mRNAs: microRNAs located on Xq27.3 associate with clear cell renal cell carcinoma. PLoS One. 2010; 5:e15224. https://doi.org/10.1371/journal.pone.0015224 PMID:21253009
