# Supplementary figures and images for: Identifying lncRNA–miRNA–mRNA networks to investigate Alzheimer’s disease pathogenesis and therapy strategy
Source: Aging (Albany NY). 2020 Feb 7;12(3):2897–920. doi: 10.18632/aging.102785 (PMC7041741; doi:10.18632/aging.102785)

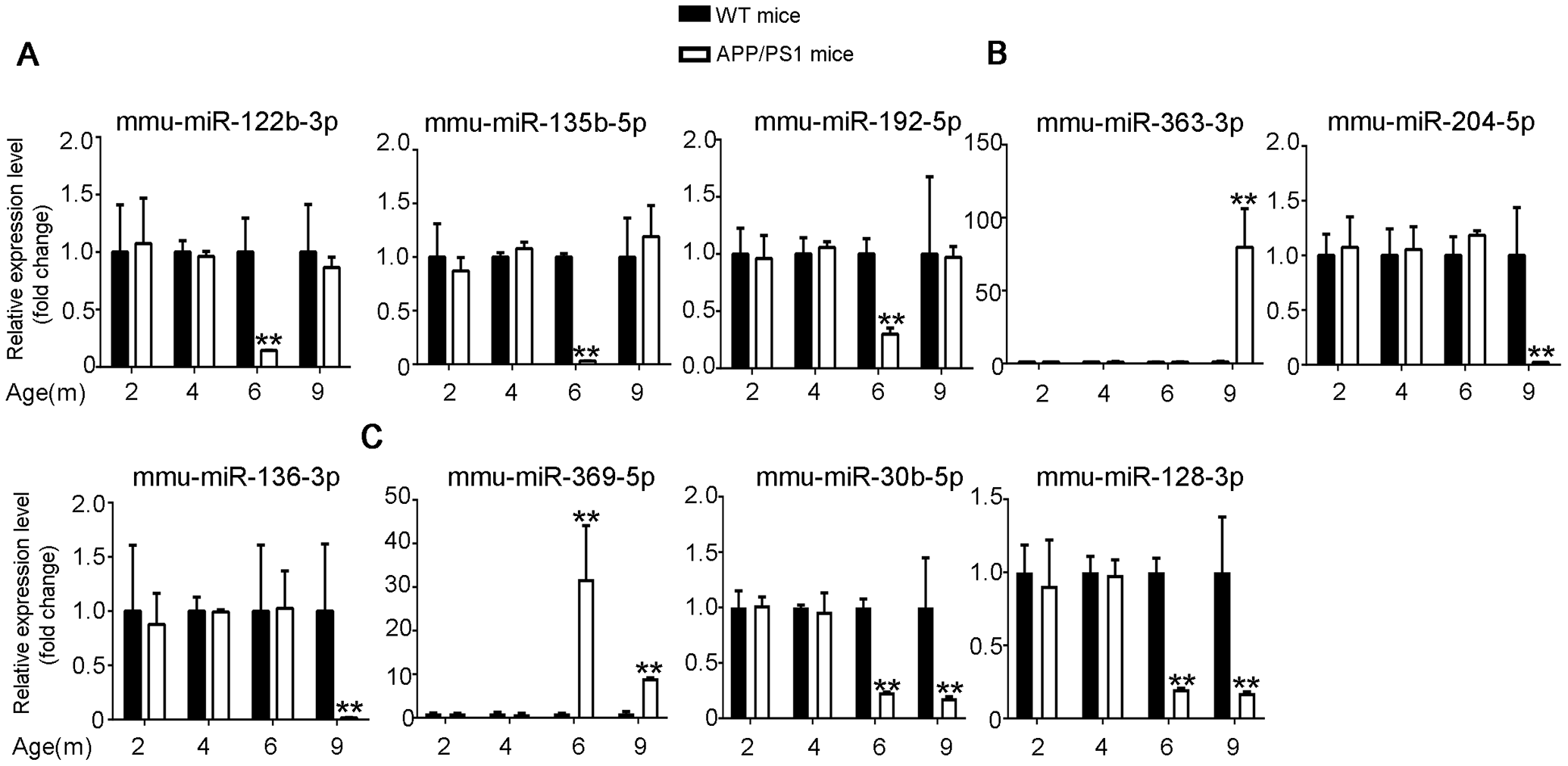

Supplement: Supplementary Table 3 [file aging-12-102785-s013..tif]
